# Announcement

**Published:** 1889-07

**Authors:** W. S. Kendrick

**Affiliations:** Atlanta, Ga.


### ANNOUNCEMENT.

A few weeks ago, owing to the death of Dr. A. B. Ashworth, it became necessary for the writer to assume control of the publication and management of this Journal. With this issue such control ceases, as a sale has been effected by which Dr. F. W. McRae, of this city, becomes its sole proprietor and manager. Dr. McRae needs no introduction to many of the readers of the JOURNAL. He graduated with distinction a few years ago in medicine, and has already reached a place and position in his profession of which his friends feel proud. As demonstrator of Anatomy in the Atlanta Medical College, his course has been marked with eminent success, and when during the winter of 1887–8, the Professor of Anatomy was ill for several months, he proved himself a master in that special department by filling his own place and the chair of Anatomy with universal satisfaction to the students. With business tact, energy and capacity, the interests of this Journal could not have fallen into more worthy and capable hands. The publication will be continued as before, and all contracts will be fully carried out. After a full investigation of all its business interests, we are fully prepared to say that the JOURNAL was never in better financial condition or on a more solid foundation, as it goes into the hands of the purchaser without a single dollar of indebtedness against it. The genial, pleasant, lamented Ashworth sleeps in the "silent city of the dead." The place he vacated at his desk cannot be better filled, and the mantle he laid aside better worn than by the new proprietor. We ask for him, on retiring, the patronage and confidence from a generous public which he so richly deserves.

W. S. KENDRICK.

*Atlanta, Ga., June 28, 1889.*

